# Lipid‐suppressed and tissue‐fraction corrected metabolic distributions in human central brain structures using 2D ^1^H magnetic resonance spectroscopic imaging at 7 T

**DOI:** 10.1002/brb3.1852

**Published:** 2020-11-20

**Authors:** Alex A. Bhogal, Tommy A. A. Broeders, Lisan Morsinkhof, Mirte Edens, Sahar Nassirpour, Paul Chang, Dennis W. J. Klomp, Christiaan H. Vinkers, Jannie P. Wijnen

**Affiliations:** ^1^ Department of Radiology University Medical Center Utrecht Utrecht The Netherlands; ^2^ Technical Medicine University of Twente Enchede The Netherlands; ^3^ MR Shim GmbH Reutlingen Germany; ^4^ Department of Psychiatry Brain Center Rudolf Magnus University Medical Center Utrecht Utrecht The Netherlands; ^5^ Department of Anatomy & Neurosciences Amsterdam UMC (location VU University Medical Center) Amsterdam The Netherlands; ^6^ Department of Psychiatry Amsterdam UMC (location VU University Medical Center)/GGZ inGeest Amsterdam The Netherlands

**Keywords:** 7 T, glutamate, metabolic imaging, MRSI, proton spectroscopy

## Abstract

**Introduction:**

Magnetic resonance spectroscopic imaging (MRSI) has the potential to add a layer of understanding of the neurobiological mechanisms underlying brain diseases, disease progression, and treatment efficacy. Limitations related to metabolite fitting of low signal‐to‐noise ratios data, signal variations due to partial‐volume effects, acquisition and extracranial lipid artifacts, along with clinically relevant aspects such as scan time constraints, are among the challenges associated with in vivo MRSI.

**Methods:**

The aim of this work was to address some of these factors and to develop an acquisition, reconstruction, and postprocessing pipeline to derive lipid‐suppressed metabolite values of central brain structures based on free‐induction decay measurements made using a 7 T MR scanner. Anatomical images were used to perform high‐resolution (1 mm^3^) partial‐volume correction to account for gray matter, white matter (WM), and cerebral‐spinal fluid signal contributions. Implementation of automatic quality control thresholds and normalization of metabolic maps from 23 subjects to the Montreal Neurological Institute (MNI) standard atlas facilitated the creation of high‐resolution average metabolite maps of several clinically relevant metabolites in central brain regions, while accounting for macromolecular distributions.

Partial‐volume correction improved the delineation of deep brain nuclei. We report average metabolite values including glutamate + glutamine (Glx), glycerophosphocholine, choline and phosphocholine (tCho), (phospo)creatine, myo‐inositol and glycine (mI‐Gly), glutathione, N‐acetyl‐aspartyl glutamate(and glutamine), and N‐acetyl‐aspartate in the basal ganglia, central WM (thalamic radiation, corpus callosum) as well as insular cortex and intracalcarine sulcus.

**Conclusion:**

MNI‐registered average metabolite maps facilitate group‐based analysis, thus offering the possibility to mitigate uncertainty in variable MRSI data.

## Graphical abstract

We use a 7 T MRI scanner along with a combination of advanced acquisition, reconstruction, and postprocessing techniques to derive metabolic distribution in deep brain structures.

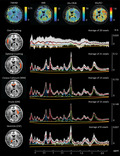

## 1 INTRODUCTION

Magnetic resonance spectroscopy (MRS) is a technique that is used to acquire in vivo brain tissue metabolite signals from single, rather large, preselected volumes that typically occupy as much as 8 cm^3^. The highly localized nature of this acquisition sharply contrasts with the ubiquitous presence of metabolites across the entire brain. Advances in MRI hardware have encouraged the development of advanced spectroscopic techniques (magnetic resonance spectroscopic imaging [MRSI]) to measure neurochemical distributions at increasingly high spatial resolutions. In contrast to single‐voxel MRS, MRSI is sensitive to diffuse and potentially heterogeneous changes in brain metabolites. As a result, MRSI has the potential to fundamentally enhance our understanding of in vivo neurochemical processes and provides a potential tool to explore cases in which pathophysiological origins of disease remain unclear or where standard structural MRI prove to be of limited diagnostic use. These new opportunities come partially as the result of the availability of ultrahigh field (>=7 T) MRI scanners that provide higher peak signal‐to‐noise ratios (SNR), as well as increased spectral dispersion to facilitate separation of overlapping metabolite resonances (for an overview, see Henning, 2018). Higher SNR improves the detection capability of lower‐SNR J‐coupled resonances such as glutamate and/or can be translated into increased MRSI resolution that can range between 2 and 5 mm^2^ in‐plane with slice thickness in the order of 10–12 mm (Bogner et al., 2012; Nassirpour, Chang, & Henning, 2018).

Contemporary studies reporting metabolite distributions measured using ultrahigh field (7 T+) MR scanners have mainly demonstrated “proof of concept” or methodological developments in specific areas such as acquisition (Gruber et al., 2017; Hangel et al., 2018; Hingerl et al., 2019; Moser et al., 2019) or data reconstruction (Nassirpour, Avdievitch, et al., 2018; Nassirpour, Chang, Kirchner, et al., 2018; Strasser et al., 2013). The translation of these techniques to the clinical environment remains hindered by sensitivity to artifacts emanating from unsuppressed water resonances (at 4.7 ppm), signal contamination by extracranial lipids (0.9–1.3 ppm; Kirchner et al., 2015), and artifacts caused by inhomogeneous *B*_1_ and *B*_0_ fields. Ongoing research focuses on overcoming some of these challenges.

Various approaches to address the issue of inhomogeneous *B*_0_ at ultrahigh field strength exist, including slice‐based shimming when requisite hardware is available (Boer et al., 2012), or postprocessing methods involving field‐map based *B*_0_ correction (Kirchner et al., 2017). Overwhelming water signals can be suppressed using saturation prepulses (Haase et al., 1985; Ma et al., 2018; Ogg et al., 1994) or inversion‐recovery based nulling of the water signal before excitation (Tkac et al., 1999). Lipid signal suppression is possible via outer volume saturation (OVS) slabs ( Henning et al., 2009); however, OVS solutions necessitate long repetition times (TR) due to restrictive specific absorption rate (SAR) limits set on radio frequency (RF) pulses. The resulting prohibitively long scan times can be mitigated with the addition of accelerated data acquisition strategies (Hangel et al., 2018; Moser et al., 2019; Nassirpour, Chang, Avdievitch, et al., 2018), replacement of OVS with postprocessing based lipid signal removal techniques (Bilgic et al., 2014), or advanced spectral‐spatial RF pulses (Hangel et al., 2015). These methods are not without their respective trade‐offs and potentially introduce unwanted artifacts or practical limitations that may restrict implementation in larger‐scale studies. Alternative hardware‐based approaches can also forgo the need for the SAR‐intensive OVS pulses through direct suppression of undesired lipid signals (Boer et al., 2015; de Graaf et al., 2018). However, such technologies can lead to collateral suppression of tissue metabolite signals that are of interest.

The aim of this study was to implement a novel combination of MRSI acquisition, reconstruction, and postprocessing methods available at our institution in order to derive lipid‐suppressed spectra at 7 T and associated metabolite values for central brain structures. We used a 7 T MR scanner to acquire high‐resolution, short echo‐time (TE) MRSI data along with an external crusher coil (Boer et al., 2015) for hardware‐based extracranial lipid signal suppression. Water suppression was achieved using a shortened chemical shift selective sequence with subject‐specific spectral‐spatial (tailored) *B*_1_‐insensitive suppression pulses (Ma et al., 2018). Advanced reconstruction and postprocessing techniques were used in conjunction with stringent data filtering based on several quality assurance (QA) metrics (Pedrosa de Barros & Slotboom, 2017) including linewidth, SNR, Cramér–Rao Lower Bounds (CRLB) and the lipid‐to‐total creatine ratio. High‐resolution anatomical images facilitated a point‐spread function (PSF) adjusted (Kirchner et al., 2015; Weber‐Fahr et al., 2002) partial‐volume correction (PVC) method that accounted for gray matter (GM) and white matter (WM) signal contributions, along with the more commonly applied correction for cerebral‐spinal fluid (CSF). Finally, registration of resulting metabolic maps to the MNI152 standard atlas facilitated the creation of high‐resolution average metabolite maps, in which macromolecular signal contributions were also accounted for (Považan et al., 2015).

## 2 METHODS

This study was approved by the medical research ethics committee of University Medical Center Utrecht, and written informed consent was obtained from all subjects. The experiments were performed according to the guidelines and regulations of the WMO (Wet Medisch Wetenschappelijk Onderzoek). Data were acquired in 23 healthy volunteers that were recruited from the general population (age 23 ± 5 years, nine females) using a 7 T MRI scanner (Phillips) equipped with a dual‐transmit head coil in combination with a 32 channel receive coil (Nova Medical). Second order image‐based shimming was performed for all acquisitions.

### 2.1 MRSI data acquisition

Suppression of extracranial lipid signals, and therefore, significant lipid contamination artifacts (Kirchner et al., 2015), was achieved using an external crusher coil (Boer et al., 2015) driven by an external amplifier capable of delivering ±10 A of current. To ensure patient safety and prevent overheating of the coil windings, 1.5 A fuse was installed between the amplifier and crusher coil. The amplifier ramp time is slow relative to the crusher coil pulse duration and the fuse burns when the integral of the current over this period exceeds 1.5 A. Image‐based calibration of the crusher coil amplifier settings is described in Figure S2. MRSI data were acquired using free‐induction decay (FID) sequence (Bogner et al., 2012) with the following parameters: 35° slice‐selective asymmetric sinc excitation pulse, TE/TR = 2.5/300 ms, Field of View FOV = 220 × 220 mm, acquisition matrix = 44 × 44, resolution 5 × 5 × 10 mm^3^, BW = 3,000 Hz, samples = 512, signal averages = 2, elliptical k‐space sampling, scan duration = 10 m 59 s, tailored spiral in‐out spectral‐spatial water suppression pulses (Ma et al., 2018). Water unsuppressed MRSI data were acquired for zeroth order phase and eddy current correction with adapted parameters: acquisition matrix = 22 × 22, resolution 10 × 10 × 10 mm^3^, signal averages = 1, scan duration = 1 min 54 s. Two adjacent 10 mm MRSI slices (20 mm slab) were acquired axially to intersect deep GM nuclei located adjacent to the ventricles while maximizing the amount of tissue acquired through the occipital lobe. This region is of interest since many MRSI studies performed at Ultra‐High Field UHF thus far have focused primarily on brain regions above the ventricles, whereas neuropsychiatric disorders (schizophrenia or psychosis, but also obsessive compulsive disorder) are believed to have origins, at least partly, in deep GM structures(Bossong et al., 2019).

In a single volunteer, a double inversion‐recovery (DIR) sequence in combination with lipid signal suppression using the crusher coil was used to acquire a measured macromolecular baseline signal (Považan et al., 2015). The slice was planned above the ventricles to minimize artifacts relating to unsuppressed water signal and *B*_0_ inhomogeneity; scan parameters were as follows: TE/TR = 2.5/1,000 ms, FOV = 220 × 220 mm^2^ resolution 30 × 30 × 10 mm^3^, BW = 6,000 Hz, samples = 512, elliptical k‐space, signal averages = 30, scan duration = 11 min 28 s. The inversion pulses were inserted within a VAPOR water suppression sequence with the first pulse at (TI_1_) 870ms before excitation, and the second, (TI_2_) 296 ms before excitation. The bandwidth of the inversion pulse was 6.7 ppm (2 kHz) with an offset of −3.7 ppm (−1110 Hz), putting the center of the inversion profile at 1 ppm. Thus, metabolite nulling was covered up to 4.3 ppm and did not affect the water resonance at 4.7 ppm. Spectral quality was evaluated visually, and a total of six voxels were isolated and averaged to generate the metabolite and lipid‐suppressed macromolecular (MM) signal. A series of nine Gaussian functions were fit to the MM signal using AMARES (jMRUI version 3.0). To minimize the degrees of freedom associated with overlapping resonances, certain MM lines were grouped (see Figure S3). Chemical shift values were as follows: MM_09 (0.94 ppm), MM_12_14 (1.29 and 1.42 ppm), MM_17_20 (1.79 and 2.04 ppm), MM_23 (2.31 ppm), MM_27 (2.75 ppm), MM_30_32 (3.02 and 3.25 ppm).

### 2.2 Imaging

A 3D Turbo Field Echo (TFE) scan (TE/TR = 2.89/8 ms, resolution = 1 mm isotropic, Field of View (FOV) = 220 × 220 × 200 mm^3^, scan duration = 6 min 51 s, flip angle = 6°) along with a shimmed dual echo GRE *B*_0_ map (delta TE/TR = 2.35/5.12 ms, FOV = 220 × 220 × 30 mm^3^, acquisition matrix = 176 × 176, resolution = 1.25 × 1.25 × 10 mm^3^, slices = 3, scan duration, 1 min 6 s) and 2D multi‐slice T1w fast field echo (FFE) image (TE/TR = 4.22/200 ms, FOV = 220 × 220 × 30 mm^3^, acquisition matrix = 176 × 176, resolution = 1.25 × 1.25 × 10mm^3^, slices = 3, scan duration, 72 s) were acquired. A reconstruction of 20 slices, each with 1 mm thickness (termed “slice”), occupying the same volume as the 20 mm MRSI slab was made on the scanner console based on the 3D‐TFE scan.

### 2.3 Reconstruction and Postprocessing

Reconstruction and processing was performed using an automatic Python‐based MRSI analysis pipeline that applied the following steps: up‐sampling of the MRSI acquisition grid by an in‐plane factor of 4 × 4 such that a single MRSI voxel became 16 voxels to match with the shimmed *B*_0_ map. Reconstruction and channel combination using coil sensitivity profiles derived from the 2D T1w FFE image (ESPIRIT method described in Uecker et al., 2014) followed by overdiscretized *B*_0_ correction based on the shimmed *B*_0_ map using algorithms described in Kirchner et al. (2017) and Kirchner et al. (2015). After reconstruction, MRSI data were down‐sampled to the acquired 44 × 44 grid using an optimized Gaussian spatial response function. These steps served to reduce near‐ and far‐reaching voxel bleeding from residual extracranial lipid signals (for details see Kirchner et al., 2015; Weber‐Fahr et al., 2002), improve overall SNR via overdiscretized noise decorrelation, and enhance spectral linewidth through overdiscretized spectral realignment (Kirchner et al., 2017). Eddy current correction (Klose, 1990) and zero‐order phase correction were performed using the unsuppressed water data, and residual water signal was removed using the Hankel Lanczos method (Cabanes et al., 2001). Automatic first‐order phase correction was achieved using a backward Yule‐Walker linear prediction autoregressive algorithm (Walker et al., 1931; Yule, 1927) on each FID signal. Total reconstruction and processing time for the steps outlined in Section 2.3 was approximately 5 min per dataset.

### 2.4 LCModel fit and metabolite map generation

^1^H MR spectral profiles were simulated for each relevant compound by NMRSIM (v. 4.6.a. Bruker Biospin) based on a pulse acquire sequence with an TE of 0.001 ms (first‐order phase correction performed during reconstruction outlined in Section 2.3). The basis set included tCho: glycerophosphocholine and choline, phosphocholine, tCr: creatine and phosphocreatine, Glx: glutamate an glutamine, taurine, myo‐inositol, glycine, glucose, tNAA: N‐acetyl‐aspartate and N‐acetyl‐aspartyl‐glutamate, gamma‐aminobutyrate, aspartate, glutathione, lactate, succinate, guanidoacetate, scyllo‐Inositol, and acetate. Chemical shift and coupling constants reported in Govindaraju et al. (2000) were implemented. All simulated spectral profiles together with parametrized MM signals that were derived from the measured MM signal served as the basis set to fit measured in vivo spectra using LCModel (Provencher, 2001; version 6.3‐1K). The amplitudes of MM components were constrained in cases where they were grouped (i.e., these peaks were bound to one another: see Figure S3). Lipid resonances (0.9, 1.3 (Lip13ab), 2.0 ppm) were simulated by LCModel. All spectra were fit between 0.2 and 4.0 ppm. The complete basis set and LCModel control parameters are included in the Supporting Information.

The metabolite values output by LCModel, along with QA data consisting of Cramer Rao Lower bounds (CRLB − %*SD*), SNR, and full‐width at half maximum (FWHM) outputs, were converted into 2D maps using custom Matlab scripts. QA maps formed the basis for binary filters that were applied to discard metabolite voxels which did not meet the following criteria: SNR > 3, FWHM < 0.15 ppm, Lip13ab/tCr < 2 or CRLB < 50%. CRLB filters based on the LCModel output were applied on a per metabolite/MM basis. Since maps were expressed as a ratio with tCr, voxels having a CRLB higher than 50% in the tCr map were removed from all metabolite maps. A caveat regarding the use of CRLB for QA is that may introduce bias in cases where metabolite values are expected to change due to disease, and absolute CRLB may be favored in these cases (for analysis see (Kreis, 2016).

Metabolic and QA maps generated from the LCModel output were regridded to the same in‐plane/in‐slice resolution as the *slice* anatomical image to mirror the reconstruction made from the 3D T1w anatomical scan (i.e., resolution from 5 × 5 × 10 mm^3^ to 1 mm^3^; matrix from 44 × 44 × 1 to 220 × 220 × 10). This step simply subdivided a single MRSI voxel into 250 voxels using nearest‐neighbor interpolation to enable high‐resolution PVC and the subsequent application of transformation matrices derived during registration of anatomical images to MNI space.

### 2.5 Partial‐volume and t1 correction

**FIGURE 1:**
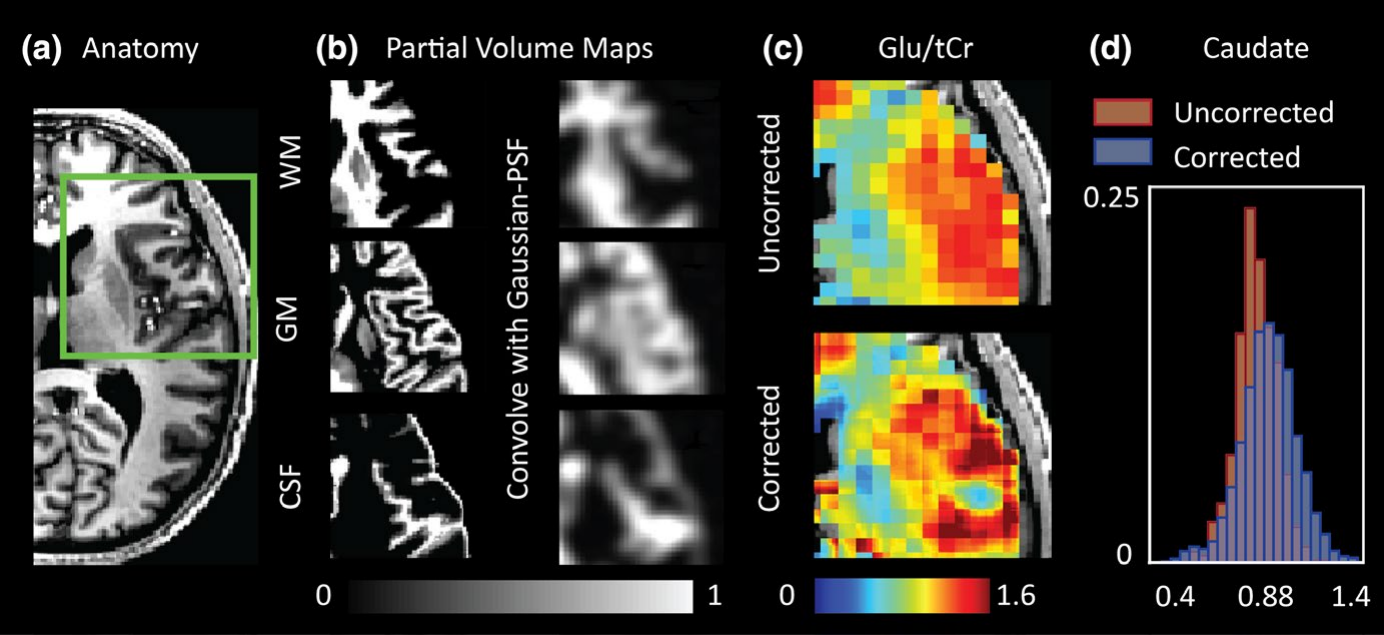
(a) T1‐weighted anatomical scan; (b) gray matter (GM), white matter (WM), and cerebral‐spinal fluid (CSF) segmentations are convolved with an appropriate point‐spread function to account for the signal dispersion (“voxel bleeding”) effects associated with reconstructing lower resolution magnetic resonance spectroscopic imaging (MRSI) data; (c) point‐spread function (PSF)‐adjusted segmentations are used for partial‐volume correction of metabolic data. A map of Glu/tCr for a single subject before and after partial‐volume and T1 correction is shown; 1D: Example probability histogram derived from MNI‐averaged Glu/tCr map in the Caudate Nucleus before and after partial‐volume correction (PVC). PVC results in an increased standard deviation due to signal dispersion throughout voxels based on partial‐volume contributions, as well as a shift to higher mean Glu/tCr in deeper GM nuclei consistent with the removal of partial‐volume effects with WM at tissue border‐zones

We extend the PVC typically done to account for CSF fraction in MRS voxels to include corrections for GM and WM tissue fractions (schematically outlined in Figure S1). To achieve this, MRSI voxel intensities were expressed as a weighted sum of pure tissue contribution. Weighting coefficients were derived from T1‐weighted anatomical images (Figure 1a) based on GM, WM and CSF segmentations (FSL: FAST) that provided the tissue's fractional volume within the voxel. For a corollary, the reader is encouraged to see similar PVC methods that have been applied to perfusion maps derived from Arterial Spin Labelling experiments (Asllani et al., 2008) and examples of spectroscopic reports reporting brain metabolite values relative to tissue fraction (Hetherington et al., 1996; Zhang & Shen, 2015). Based on prior knowledge regarding the MRSI PSF and Gaussian response function applied during reconstruction (see Section 2.3 and Kirchner et al., 2015, 2017), anatomical segmentations were convolved with an appropriate PSF to mimic the signal bleeding effects inherent to the MRSI acquisition (see figure 1b and methods in Weber‐Fahr et al., 2002). For generation of the PSF, the k‐space shutter applied during acquisition, along with the acquisition matrix of the anatomical data used for PVC, was taken into account. The steps used to generate the PSF are elaborated on in Figure S4. The zero‐padding associated with the elliptical k‐space shutter and overdiscrete reconstruction yielded a PSF with a 8.75 mm FWHM. Using this information, metabolite values within a voxel were then redistributed (within the same occupied volume) based on point‐spread adjusted tissue‐fraction and pure tissue metabolite values (see Figure S1). Due to low metabolite abundance, the CSF contribution to the total metabolite signal was considered to be negligible. Pure tissue contributions were estimated by linear regression of measured metabolite values that remained after QA filtration against the normalized tissue fraction of the voxel (GM fraction divided by the sum of the GM and WM fractions) and extrapolating to a GM fraction of 1 (pure GM) or 0 (pure WM; Gasparovic et al., 2011; Hetherington et al., 1996): see Figure S1. Partial‐volume corrected maps were corrected for steady‐state T1 relaxation effects based on tissue‐specific T1 values reported at 7 T by Xin et al. (2013). T1 values are provided in Table S1.

### 2.6 Normalization of MRSI data to standard space

**FIGURE 2:**
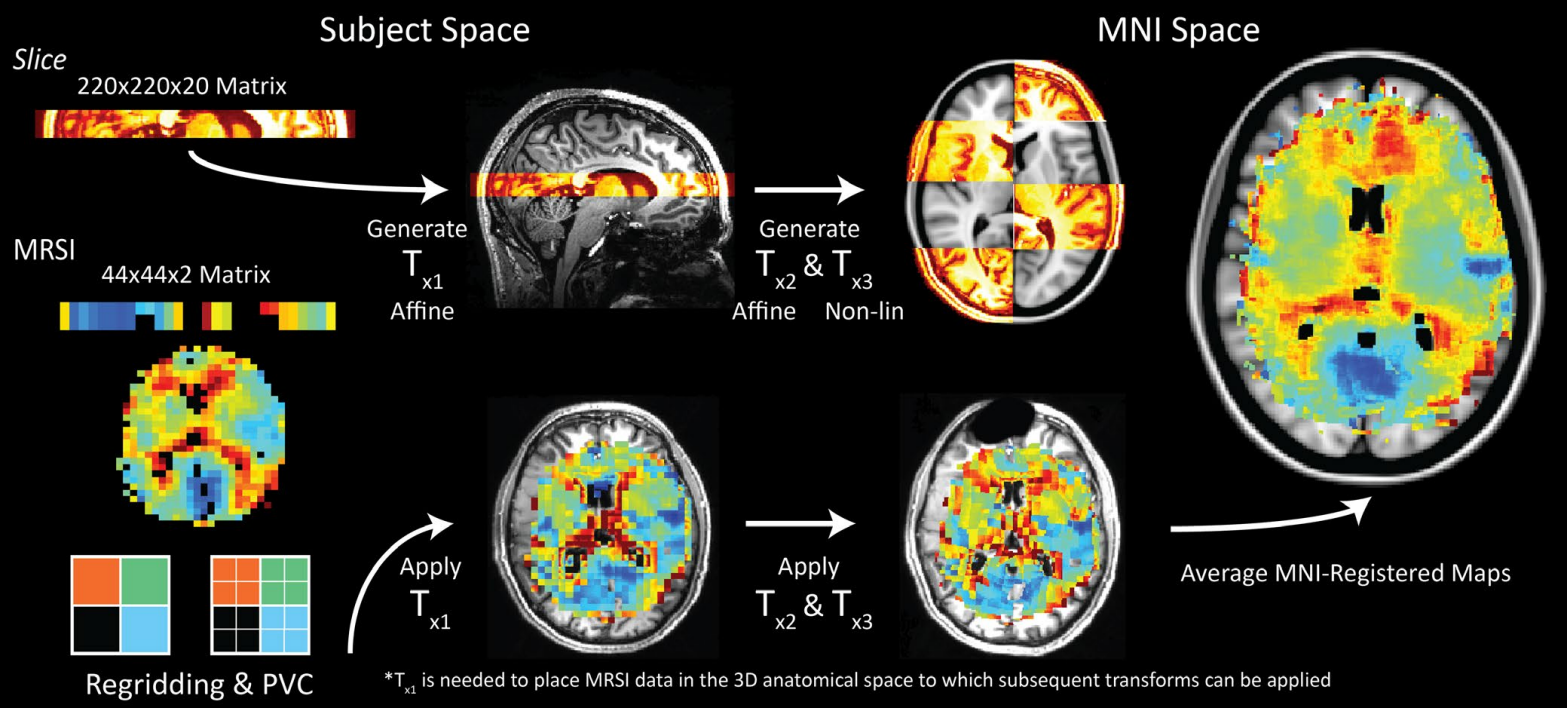
Top: An anatomical “slice” is reconstructed on the scanner console from the whole‐brain 3D T1‐weighted anatomical scan. This slice occupies the same volume as the magnetic resonance spectroscopic imaging (MRSI) acquisition. The slice is registered back to subject space (T1w image) using an affine transformation. The T1w image is then registered to MNI space via affine and nonlinear transformations; Bottom: Metabolic maps (unsmoothed tCho/tCr from a single subject is shown) are resampled to the same resolution as the reconstructed slice (nearest‐neighbor interpolation). The transformation matrices (T_x1–3_) derived by registration of anatomical data from subject to MNI space are applied to the resampled metabolite maps. MNI‐registered MRSI data is averaged in MNI space. The MNI‐registered tCho map based on 23 subjects is shown (right). MNI data depicted here have not been smoothed. Warmer colors (yellow‐red) depict choline‐rich white matter regions. The effect of partial‐volume correction is notable in cerebral‐spinal fluid‐rich regions (blue) such as the posterior interhemispheric fissure and sulci.

Partial‐volume corrected metabolite maps were normalized to MNI space by applying three transformation matrices generated using the FSL software library (Smith et al., 2004; Figure 2). The first transformation (*T*_X1_) was derived from the rigid registration (FSL: FLIRT; Jenkinson & Smith, 2001) of the reconstructed *slice* image to the 3D T1w scan. Next, brain extracted anatomical images (optiBET shell script; Lutkenhoff et al., 2014) were registered to the 1 mm MNI152 atlas via rigid (FLIRT) and then nonlinear transformations (FSL: FNIRT; Andersson et al., 2010) to generate *T*_X2_ and *T*_X3_, respectively. Finally, transformations were applied, in order, to create MNI‐registered metabolite and QA maps. These maps were averaged, and the data density (i.e., how many subject datasets overlapped at a particular voxel coordinate) was calculated on a voxel‐wise basis. To examine regional metabolite values, MNI masks were chosen based on their spatial correspondence with the metabolic data contained in the average maps; GM segmentations: accumbens, caudate, pallidium, putamen, thalamus, intracalcarine (IC) cortex, insular cortex, and total cerebral cortex; WM segmentations: corpus callosum, thalamic radiation, and total WM.

## 3 RESULTS

Despite the “blurring” of structural data resulting from convolution with the Gaussian PSF (Figure 1b), considerable differences between corrected and uncorrected metabolite maps were evident (Figure 1c). Specifically, the effects of PVC were most obvious in regions containing CSF where metabolite signals were redistributed to neighboring tissues, as well as in regions containing transitions between GM and WM. The later effect was most notable for metabolites such as Glu/Glx and tCho. An representative example showing the change in Glu/tCr distribution for the caudate nucleus in a single subject is provided in Figure 1d. Here, WM signals located at regional border zones are redistributed outside of the caudate nucleus resulting in an increase in the mean Glu/tCr value. Accounting for variable GM tissue fraction also result in broadening of the distribution shape. Our work supports the findings of Maudsley et al., (2009) and Goryawala et al. (2016) that have reported regional variability of these metabolites in healthy subjects using MRSI methods at 3 T.

**FIGURE 3:**
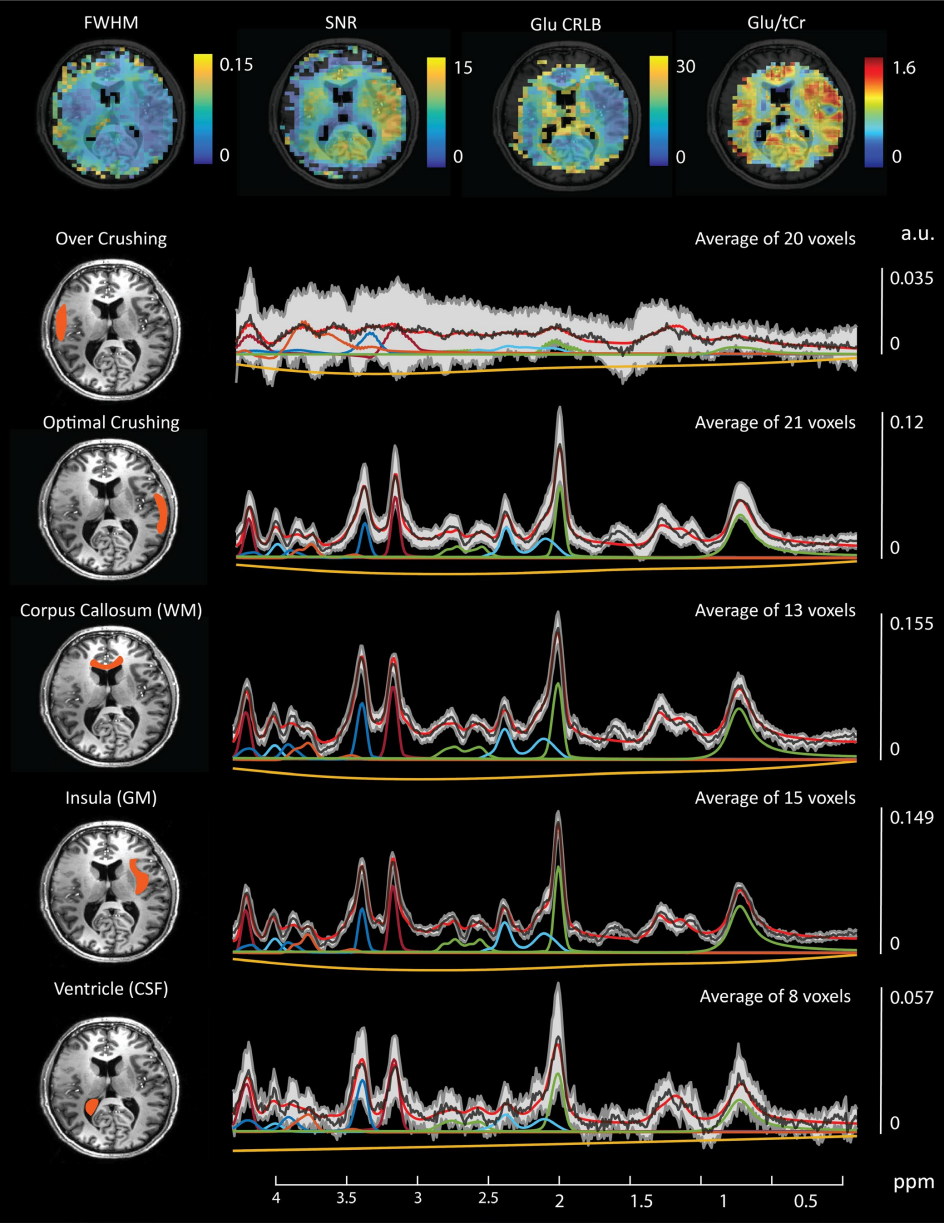
Top: Example single‐subject quality assurance (QA) maps. Voxels with signal‐to‐noise ratios (SNR) values lower than 3, full‐width at half maximum (FWHM) values higher than 0.15 ppm, and Cramér–Rao Lower Bounds (CRLB) values higher than 50% are removed. Thresholded QA maps are binarized and used to filter corresponding metabolite maps. A QA filtered Glu map is shown Top‐Right. Overlay of metabolite maps with anatomical data allows precise delineation of specific regions of interest (ROI) for spectral plotting. Average spectra for example ROIs (left) are denoted by the black line with standard deviations defined by the gray shaded area. Corrections for inhomogeneous B_1_ were not performed so spectral signal intensity variations may show artificially large standard deviations (gray shaded area). Average fit values for selected metabolites are shown for each ROI: MM09 (green centered at 0.9 ppm) tNAA (green centered at 2.0 ppm), Glx (light blue), tCr (red), tCho (dark blue), mI + Gly (orange), fit baseline (yellow), total fit (red), and average signal (black)

**FIGURE 4:**
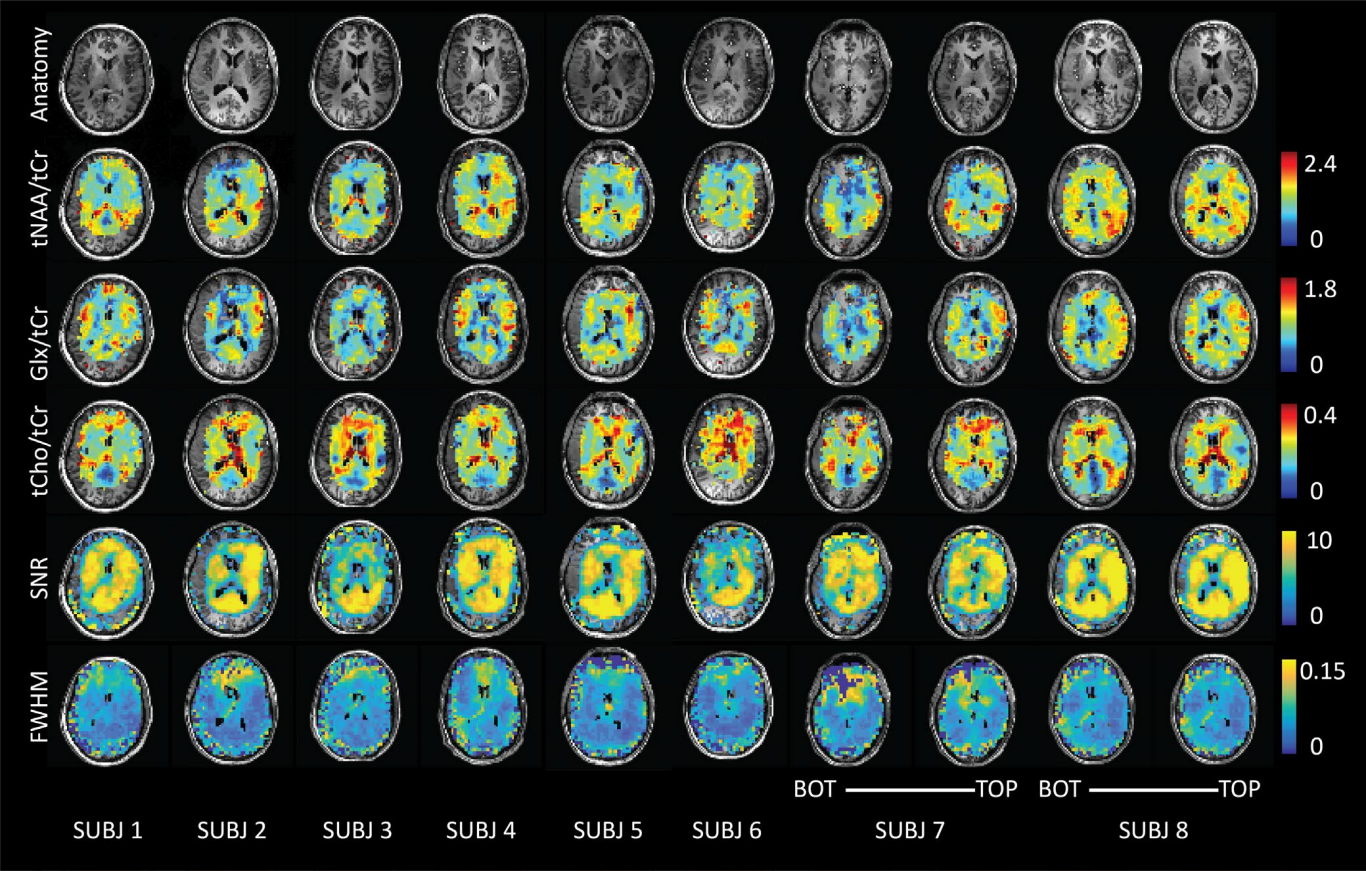
Partial‐volume corrected metabolite data of eight subjects are shown for tNAA, Glu, and tCho along with corresponding signal‐to‐noise ratios (SNR) and full‐width at half maximum (FWHM) maps derived from the LCModel “miscellaneous output.” Both the bottom and top slices are shown for subject numbers 7 and 8

An indication of the amount of data removed for various QA filters is provided in Table S2. Due to asymmetries in the lipid suppression gradient, cortical metabolite signals subject to the suppression field were consistently filtered out during the QA process. The contrast in quality between spectra originating in “over crushed” versus “optimally crushed” cortical regions is shown in Figure 3. Overcrushed cortical regions were generally filtered out due to low SNR or high FWHM (Figure 4).

**FIGURE 5:**
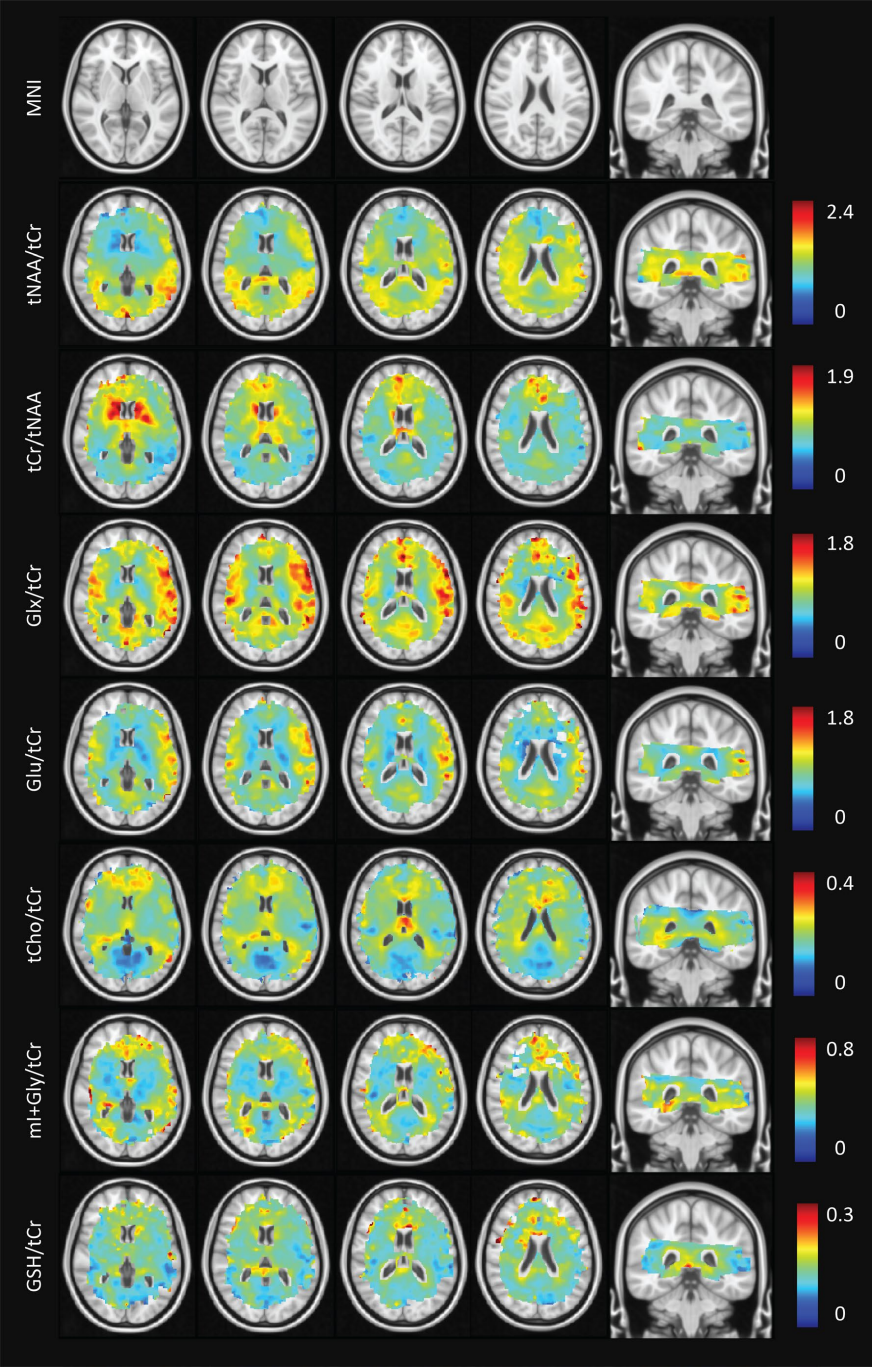
MNI‐averaged metabolite maps derived from 23 subjects. Voxels containing less than three data points have been removed

Average metabolite maps are shown in Figure 5 and are reported for applicable MNI segmentations in Table 1. The known differences in Glu/tCr (or Glx/tCr) between GM and WM were reproduced (Goryawala et al., 2016). Elevated Glu/tCr levels were measured in cortical structures (insula, IC, and cerebral cortex), with reduced levels found in WM and subcortical structures in line with results reported using single‐voxel MRS at 7 T (Marjanska et al., 2012). These distributions are consistent with the role of Glu in energy production and neurotransmission. Higher tCho/tCr levels were seen in WM as compared to GM. The levels in the occipital lobe, the insular and IC cortex, and the cerebral cortex were likely reduced due to negligible signal contributions from CSF. The levels of tCho/tCr reflect soluble membrane components (Lin et al., 2005), and since much of GM is occupied by microvasculature (and therefore blood, rather than cell membrane components), WM tCho/tCr levels were in line with expectations. In addition, the regional heterogeneity with elevated levels in frontal regions shown in our maps supports single‐voxel results presented by Pouwels & Frahm (1998) and Baker et al. (2008). Levels of tNAA/tCr in the nucleus accumbens, caudate, pallidum, and putamen were low as compared with the thalamus and cortical GM structures. The low ratio of tNAA/tCr in the accumbens supports findings reported by Xi‐Long et al. using single‐voxel MRS at 3 T (Liu et al., 2017). High levels are expected in cortical structures since NAA, the dominant resonance around 2.0 ppm, is considered a marker of viable neurons and neuronal density is highest in cortical GM regions. The difference in ratios between cortical/thalamic regions and basal ganglia may be explained by their varying physiological functions and structural organization. The cortical GM is arranged in layers, while the basal ganglia are composed of distinct GM masses (Lanciego et al., 2012) with integrative control functions. The combined map of mI + Gly/tCr showed higher ratios in the corpus callosum and neighboring WM structures. Lower values were seen in cortical/insular structures. While ratios were higher in low‐frontal regions of the brain, it is possible that this region was affected by inhomogeneous *B*_0_ around the nasal cavities, residual water signal around 4.7 ppm or low data density, and therefore greater uncertainty stemming from intersubject variability. Nevertheless, GM versus WM differences in mI + Gly/tCr were apparent, which is consistent with previous single‐voxel experiments (Baker et al., 2008) and metabolic maps acquired at 9.4 T (Nassirpour, Chang, & Henning, 2018).

**TABLE 1:** Average metabolite ratios across different brain regions

	Regional metabolite levels (ratios over tCr)					
	Glu	Glx	tCho	tNAA	mI + Gly	GSH
MNI region						
Corpus callosum	0.73 ± 0.13	0.90 ± 0.16	0.23 ± 0.04	1.26 ± 0.22	0.45 ± 0.09	0.17 ± 0.04
Thalamic radiation	0.68 ± 0.11	0.86 ± 0.14	0.23 ± 0.03	1.0 ± 0.17	0.39 ± 0.10	0.16 ± 0.03
White matter	0.72 ± 0.14	0.88 ± 0.17	0.22 ± 0.04	1.27 ± 0.20	0.41 ± 0.11	0.15 ± 0.04
Nucleus accumbens	0.72 ± 0.07	1.0 ± 0.07	0.24 ± 0.03	0.54 ± 0.07	0.42 ± 0.15	0.21 ± 0.07
Caudate	0.66 ± 0.12	0.85 ± 0.16	0.22 ± 0.03	0.84 ± 0.18	0.37 ± 0.08	0.18 ± 0.05
Pallidum	0.58 ± 0.10	0.71 ± 0.12	0.18 ± 0.01	0.85 ± 0.19	0.31 ± 0.06	0.15 ± 0.04
Putamen	0.78 ± 0.10	0.97 ± 0.13	0.19 ± 0.02	0.84 ± 0.14	0.32 ± 0.06	0.15 ± 0.03
Thalamus	0.75 ± 0.10	0.93 ± 0.12	0.22 ± 0.03	1.07 ± 0.12	0.36 ± 0.09	0.15 ± 0.02
Intracalcarine sulcus	0.89 ± 0.11	1.1 ± 0.16	0.13 ± 0.02	1.36 ± 0.21	0.35 ± 0.08	0.12 ± 0.02
Insular cortex	0.98 ± 0.12	1.2 ± 0.16	0.21 ± 0.04	1.12 ± 0.15	0.38 ± 0.10	0.14 ± 0.03
Cerebral cortex	0.97 ± 0.20	1.2 ± 0.27	0.17 ± 0.05	1.13 ± 0.28	0.41 ± 0.16	0.15 ± 0.06

**FIGURE 6:**
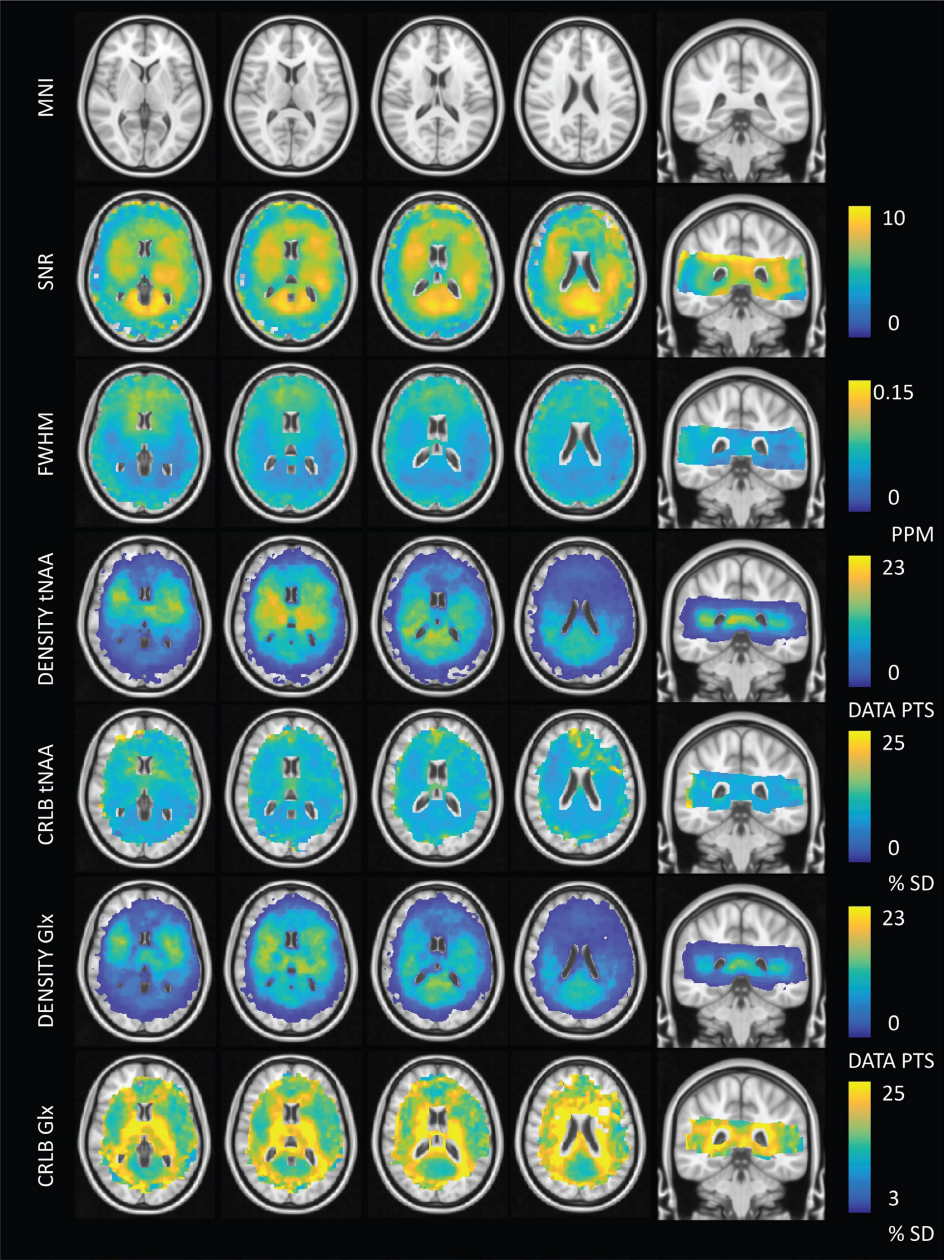
Quality assurance (QA) maps averaged across 23 subjects. The signal‐to‐noise ratios (SNR) and full‐width at half maximum (FWHM) data are output for each individual LCModel fit and show an inverse relationship between signal intensity and linewidth as expected. SNR values less than 3 and FWHM values greater than 0.15 ppm have been filtered out. Density maps for tNAA and Glx are provided. In general, data density is high in central regions of the brain at the location of subcortical grey matter (GM). Corresponding Cramér–Rao Lower Bounds (CRLB) maps are provided illustrating regions of variable fit confidence. This is notable for Glx, where the CRLB is higher within white matter (WM) regions. This is in agreement with the fact that Glx is approximately 50% less abundant in WM versus GM. Lower peak SNR leads to increased fit uncertainty and higher CRLB. CRLB values greater than 50% have been removed

In addition to metabolic information, QA and data density information was evaluated in MNI space. As expected, an inverse spatial relationship was observed between SNR and linewidth FWHM (with regions of low SNR displaying broader linewidths; see Figure 6). This was likely a result of inconsistencies in shimming, variations in the $B_{1}^{+}$ field, or possible effects relating to the lipid suppression field. Reduced SNR with increased FWHM was observed at frontal regions of the brain, reflecting inhomogeneous *B*_0_ arising from susceptibility effects around the nasal cavities. This trend was consistent with higher CRLB values for tNAA at frontal regions of the brain. In comparison, CRLB values for Glx were higher along WM tracts (Figure 6). Glx consists of low SNR metabolites with low WM abundance relative to GM. These factors contribute to greater fit uncertainty and relatively higher CRLB within WM regions. Density maps highlight increased information sampling at central regions of the brain consistent with acquisition of data at the level of the subcortical nuclei.

## 4 DISCUSSION

One of the major promises of MRSI is the ability to detect physiological changes in disease in the absence of structural/anatomical markers. While single‐voxel spectroscopic measurements might be considered the gold standard in terms of reliability of data, evolving metabolic mapping techniques provide opportunities for evaluating the metabolic state of the entire brain. Our focus on artifact control, PVC, QA filtration, and data aggregation from multiple subjects has permitted mapping of metabolite ratios metabolites such as tNAA, Glx, and tCho at deeper regions of the brain adjacent to the ventricles; areas of interest for studying metabolic alterations linked to psychiatric disorders (Bossong et al., 2019; Merritt et al., 2016).

An MNI‐registered metabolic brain atlas based on pooled MRSI data was first presented by Maudsley et al. (2009) who mapped distributions of certain human neurochemicals using 3 T‐EPSI acquisitions. Our work diverges in several important aspects. The use of a crusher coil for direct lipid suppression, instead of postprocessing or inversion methods for lipid signal removal, meant that we could acquire MRSI data without the risk of manipulating metabolite signals adjacent to lipid resonances, or residual lipid contamination due to imperfect inversion pulses. Furthermore, in our approach, the signals of lactate and macromolecules stay intact and can be taken into consideration during metabolite fitting. Another key difference is our use of the coregistered high‐resolution anatomical information for PVC. While PVC of spectroscopic data is not a novel concept, a large majority studies correct for only CSF, whereas we consider tissue‐specific signal contributions arising from WM and GM at 1mm isotropic resolution. Finally, the higher SNR at 7 T has allowed us to generate voxel‐wise maps of low signal resonances such as Glu, where seminal work performed at 3 T relied on ROI‐based analysis for the derivation of similar information (Goryawala et al., 2016).

Our results complement contemporary (UHF) MRSI reports that tend to focus on brain regions located above the ventricles (Bogner et al., 2012; Hangel et al., 2018; Nassirpour, Chang, & Henning, 2018); likely as a way to reduce sensitivity artifacts stemming from inhomogeneous *B*_0_ or unsuppressed water while showing the potential of new techniques. Aside from acquisition at optimal brain regions, these innovations can be showcased by optimizing data quality through increased SNR using longer TR, removing (or excluding during metabolite fitting) lipid signals between 0 and 1.8 ppm, or acquiring at higher spatial resolutions at the cost of SNR and scan time. Our use of tailored water suppression pulses and a crusher coil in combination with a short TE/TR acquisition allowed us to obtain reasonably high‐resolution, lipid‐suppressed MRSI data within 11 min. Clinically acceptable scan times are paramount for widespread adoption of MRSI due to the general requirement of additional anatomical and functional imaging in a typical patient scan slot. Current efforts to offset scan time using fast MRSI techniques show great potential and include accelerated data acquisition via echo planar readout (EPSI) gradients (Coello et al., 2018; Otazo et al., 2007) or alternate k‐space sampling strategies combined with advanced reconstruction methods (Ma et al., 2017; Nassirpour, Chang, Avdievitch, et al., 2018). The advantages and limitations of various fast MRSI techniques have been thoroughly reviewed by Vidya Shankar et al. (2019).

We improve upon previously reported MRSI data registration methods through the use of the high‐resolution “slice” reconstruction as a surrogate for the MRSI data (Figure 2). This reduced mis‐registration errors and minimized additional uncertainties relating to intersubject variability (Pouwels & Frahm, 1998). A consequence of spatially averaging metabolite maps at the resolution of the 1 mm MNI152 atlas was an apparent increase in signal contrast between brain regions; particularly in striatal/thalamic regions of the brain where data density was high. This is a point of significance for future group‐level comparisons seeking to identify regional differences in small brain structures. A possible drawback of our approach is that we cannot take into account subject motion occurring between the T1w and MRSI scans.

### 4.1 Considerations

Our acquisition protocol was guided by the preference toward reliable signal, and this often meant that some tissue signals were overcrushed. This was exacerbated by factors relating to asymmetries in the crushing field (Figures 3 and 4) or subject head size and/or positioning within the lipid suppression coil itself. In general, “overcrushed” spectra are easy to identify and remove via QA; however, distinguishing partially crushed signals from low signal intensity due to low peak height stemming from regional metabolite abundance may be less trivial. In these cases, comparison with reference spectra from a whole‐brain spectral atlas or more advanced deep‐learning spectral selection techniques may be of use. Considering this, future coil design iterations will focus on greater control of the lipid suppression field through modular design and the use of novel winding patters (Huijing et al., 2019) as well as multi‐channel capabilities for regional control of the suppression fields. Reconstruction (Bilgic et al., 2014) and postprocessing based lipid removal methods can provide alternative pathways for lipid suppression while conserving MRS signals at the edge of the cortex; however, these methods may have drawbacks relating to signal removal of nearby metabolite resonances (Haupt et al., 1996), or overlapping lactate or macromolecular signals (i.e., around 1.2 and 1.4 ppm). Second, while it is advised to include subject‐specific MM signals into fitting protocols for optimal signal quantification (Cudalbu et al., 2012), scan time constraints and the need to generate a new basis per subject left this approach unfeasible. Considering the low resolution of the DIR acquisitions (30 × 30 × 10 mm^3^) along with reports that a general MM baseline is suitable for GM and WM at 7 T (Benoît et al., 2014; Snoussi et al., 2015), it was concluded that high‐quality data from a single volunteer was a reasonable compromise. In line with current literature, we chose to use tCr as an internal reference (Posse et al., 2013). Using ratios divides out larger variations associated with inhomogeneous *B*_1_ transmit/receive fields and coil loading. An important caveat specific to ultrahigh field studies is that the inhomogeneity of the excitation pulse across brain regions can lead to variable T1 weightings between metabolites. Absolute quantitation using the water reference along with using additional *B*_1_ correction steps during postprocessing (requiring *B*_1_ mapping scans) could further improve the accuracy of our reported metabolite ratios. A further source of error may arise under conditions where metabolite peak heights show *B*_1_ dependent T1 weightings. While we corrected for bulk T1 effects per metabolite, we were not able to correct for inhomogeneous relaxation constants between hydrogen‐containing molecular subgroups or between different brain regions (An et al., 2017). This is an issue that is further compounded when considering combined metabolites (tCho, tNAA, Glx, or tCr). The issue of inhomogeneous excitation profiles could be addressed by the implementation of parallel RF transmit systems.

On a related note, normalization to tCr assumes a homogeneous distribution across brain regions and can therefore introduce errors under circumstances where tCr levels in the brain have changed due to disease. Disease‐related changes in metabolite distributions may also negatively impact the accuracy of metabolite signal scaling during PVC since we currently assume that metabolite ratios for pure tissues remain stable. For this reason, caution is advised if using PVC for areas in which structural changes have occurred (e.g., brain tumors or multiple‐sclerosis lesions). It should be noted, however, that many neurological diseases do not result it visible lesions. These include psychiatric disorders, or prodromal phases of disease in which “normal‐appearing” tissues begin to undergo biochemical changes. While errors in calculating the linear tissue‐fraction dependent metabolite concentrations may occur, the PVC does not change local average metabolite values. Therefore, elevated or reduced values will remain, and subtle differences may perhaps be highlighted after correction. Nevertheless, investigations into nonlinear relationships between tissue‐fraction and metabolite values are warranted for pathological cases.

In general, our measured metabolite values were lower than those reported in literature. Possible sources for these differences are as follows: (a) we include MM signals that overlap with metabolite resonances; (b) we perform T1 and PVC for GM and WM tissue contributions, which is uncommon for MRSI studies. In some cases, the correction for the T1 of tCr reduces metabolite ratio values; (c) we use a relatively short TR of 300 ms that may introduce T1 weightings particularly for metabolites with long T1s. Based on our flip angle and repetition time, our sequence was optimized to give the highest SNR per unit time for metabolites with T1 values in the range of 1,500–2000 ms; (d) differences in metabolite fitting software or LCModel control file parameters are known to have a significant impact on peak fitting results (Bhogal et al., 2017).

## 5 CONCLUSION

In this work, we present in vivo metabolite levels for central brain structures obtained by combining several methodological advances, including a crusher coil to suppress the high intensity extracranial lipid signals, advanced reconstruction techniques, PVC based on high‐resolution anatomical images, and normalization to a standard space. Our acquisition and processing pipeline facilitates group‐level 7 T MRSI analysis and provides an infrastructure to which data from ongoing and future studies using comparable acquisition techniques can be easily added. Given the spatial coverage, values reported herein may be of interest for future studies investigating WM and subcortical GM physiology. Registration of MRSI data to standard space opens the possibility to compare and correlate 7 T MRSI metabolic data with any parameters normalized into the same space.

## CONFLICT OF INTEREST

The authors have no conflicts of interest to disclose.

## AUTHOR CONTRIBUTIONS

A.A.B. contributed to experimental design, acquisition, data analysis and interpretation of data, and writing of the manuscript. T.A.A.B., L.M., and M.E. contributed in data acquisition and analysis. S.N. and P.C. contributed to data analysis. D.W.J.K., C.H.V., and J.P.W. contributed in conception and design as well as revision and approval of the manuscript.

## Supporting information

Supplementary Material

## Data Availability

The data that support the findings of this study are available from the corresponding author upon reasonable request.
